# Advice and Frequently Asked Questions (FAQs) for Citizen-Science Environmental Health Assessments

**DOI:** 10.3390/ijerph15050960

**Published:** 2018-05-11

**Authors:** Timothy M. Barzyk, Hongtai Huang, Ronald Williams, Amanda Kaufman, Jonathan Essoka

**Affiliations:** 1 National Exposure Research Laboratory, U.S. Environmental Protection Agency, Research Triangle Park, NC 27711, USA; williams.ronald@epa.gov; 2 Program on Reproductive Health and the Environment, University of California, San Francisco, CA 94143, USA; Hongtai.Huang@ucsf.edu; 3 Office of Air Quality Planning and Standards, U.S. Environmental Protection Agency, Research Triangle Park, NC 27711, USA; kaufman.amanda@epa.gov; 4 U.S. Environmental Protection Agency, Region III, Philadelphia, PA 19103, USA; essoka.jonathan@epa.gov

**Keywords:** citizen science, environmental health assessment, decision analysis, local stakeholders, cumulative impacts, environmental justice

## Abstract

Citizen science provides quantitative results to support environmental health assessments (EHAs), but standardized approaches do not currently exist to translate findings into actionable solutions. The emergence of low-cost portable sensor technologies and proliferation of publicly available datasets provides unparalleled access to supporting evidence; yet data collection, analysis, interpretation, visualization, and communication are subjective approaches that must be tailored to a decision-making audience capable of improving environmental health. A decade of collaborative efforts and two citizen science projects contributed to three lessons learned and a set of frequently asked questions (FAQs) that address the complexities of environmental health and interpersonal relations often encountered in citizen science EHAs. Each project followed a structured step-by-step process in order to compare and contrast methods and approaches. These lessons and FAQs provide advice to translate citizen science research into actionable solutions in the context of a diverse range of environmental health issues and local stakeholders.

## 1. Introduction

Cumulative risk assessments (CRAs), health impact assessments (HIAs), cumulative impacts, environmental justice (EJ) scores, and related aggregators of environmental health information capture a range of factors that positively and negatively impact humans, animals, ecosystems, and the general state of the natural environment [1,2,3,4]. Each approach has benefits and limitations and typically requires both quantitative measurements and qualitative weights of evidence [5,6,7]. For example, the Centers for Disease Control and Prevention (CDC) lists six different types of health assessments on their website (https://www.cdc.gov/healthyplaces/types_health_assessments.htm).

For the scope of this paper, an *Environmental Health Assessment* (EHA) is a methodical evaluation of air, water, and soil pollution impacts on human health and the environment in the context of all possible health determinants, and the subsequent development of risk reduction actions. However, the term *health* is widely encompassing, ranging from cancer clusters, asthma attacks, cardiovascular emergency room visits, and species degradation, to psycho-social stress, quality of life, and community vulnerability [8]. The *surrounding environment* includes anything the receptor can see, feel, touch, breathe, hear, taste, or otherwise sense and react to. The reason for this level of openness is to accommodate the variety of stressors (negative contributors) and salutary factors (positive contributors) in local areas and communities [9,10,11], as well as the range of local stakeholders associated with environmental health [1,12]. 

For groups with limited human, technical, and financial resources, EHA efficiency and effectiveness are important considerations. Publicly available EHA approaches do not currently exist that would mandate enforcement and compliance actions. Stakeholders (people directly or indirectly involved or affected) could include community residents and organizations, academics, commercial interests, and local/federal governments [1,13]. Yet no standardized approach exists to transfer results from one entity to another, so that each entity is allowed a subjective decision to accept or reject the findings of another, not only between like entities, such as two separate community groups or state agencies, but especially between different entity types. For example, government agencies are under no legal obligation to change policies based on citizen science measurements [1,14,15]. 

The network of partners who develop EHAs can be more influential in promoting change than the quantitative results themselves [6,16]. This is an important consideration for inexperienced groups who hope that quantitative results alone will promote improvements in human and environmental health. Sometimes the process is more impactful than the utility of the data collected [17]. This paper presents two citizen science EHAs and reflects on a decade of collaborations. This work culminated in three lessons learned and a set of Frequently Asked Questions (FAQs), focused on public communication for citizen science projects. This work is not intended as a framework or conceptual approach, a step-by-step method, or a roadmap. It provides advice to translate research into action, manage expectations, facilitate understanding, and target resources.

## 2. Methods

Two locally-based projects and over a decade of collaborative input contributed to this work (Table 1). The two projects included partners from government, community, academia, and non-profit organizations. State and local agencies and commercial interests were not included; however, they have worked with each respective partner outside of these efforts. Partners represented different types of organizations and geographic scales of interest, from residents focused on their neighborhood, to regional agencies covering multiple states, to federal researchers hoping to transfer successful methods across the country.

Projects were led by partnerships that encountered professional and inter-personal challenges. This is the nature of real-world, non-regulatory assessments and one of the pivotal reasons for this research: to help mediate the scientific, personal, and political aspects of environmental health decision-making. Both projects, and several from Table 1, followed an identical, structured, iterative process [1] with rigorous documentation, allowing compare-and-contrast among the different projects. These findings led to development of the lessons learned and FAQs. Steps included:

1. Form partnership and identify stakeholders
2. Define goals, objectives, and hypotheses
3. Identify environmental health stressors and salutary factors
4. Collect data, topic-expert knowledge, and local input
5. Rank environmental health stressors and salutary factors
6. Identify risk mitigation strategies
7. Collect information on technical, financial, and human resources for mitigation
8. Prioritize risk mitigation strategies
9. Plan post-project long-term goals
10. Measure success using agreed-upon metrics

Newark, Newport News, and several projects from Table 1 maintained regular monthly meetings with the entire partnership throughout the course of each project, with interim meetings between smaller groups to maintain specific elements. Rigorous meeting notes and summaries were compiled for each project, with challenges and limitations being a primary discussion topic. More than 40 meetings were held between the Newark and Newport News projects alone, and over 100 meetings total for all projects, with participants having equal time to voice topics and concerns. Documentation for each assessment step, including meeting notes, summary tables, data outputs, and proposed approaches, were then compared and contrasted across projects in order to summarize the primary challenges (i.e., lessons learned) and research questions (i.e., FAQs). 

While FAQs were in development with several of the earlier projects listed in Table 1, they came to fruition during the Newark project primarily. That project had significant challenges with interpretation and communication, with multiple meetings to address them. For example, the community group expressed concerns that local health issues were not being addressed and that sensor measurements would not influence policy, whereas Environmental Protection Agency (EPA) sensor experts stated that the research design and sensor capabilities were unable to directly address these concerns and that a local scale characterization of air quality concentrations was the extent of the research, in addition to the local training on sensor capabilities. Multiple facilitated discussions led to a communications plan to address this disconnect. After the project, remaining partners synthesized the FAQs presented here to help increase transparency for future projects. These FAQs were then applied to the Kansas City project (Table 1) during its research design, including with the Community Action Group (CAG) liaison, and were also vetted through EPA citizen science communications experts and multiple EPA Regional personnel who work directly with communities on HIAs. 

### 2.1. Newark Project

#### 2.1.1. Location Description

The Ironbound is a four square mile unincorporated community within the city of Newark, New Jersey, largely populated by Portuguese, Spanish, and Latin American immigrants and their descendants. The area’s name refers to the proliferation of railway and metalworking industries. It is bounded by highways, waterways, and lies adjacent to the Newark International Airport and the Port of Newark/Elizabeth, making it a hub of multi-modal freight transport. The population of more than 50,000 represents one of the most densely populated and diverse communities of the city. Two-thirds of the population are foreign-born, and 75% of those over the age of five speak a foreign language at home, typically Portuguese or Spanish. Educational attainment is relatively low with 55% of those over 18 not having a high school diploma. Census tracts range from 25% to 55% of households below the poverty level. Hundreds of tenants from three public housing complexes (comprised of more than 700 units) live near industrial land uses, hazardous and Superfund sites, and major highways. More information on the Ironbound community and this project can be found in Kaufman et al. [18].

#### 2.1.2. Partners, Goals, Objectives

Local, state, and federal partners were represented in this project. The Ironbound Community Corporation (ICC) was founded in 1969 with the mission to, “engage and empower individuals, families, and groups in realizing their aspirations and, together, work to create a just, vibrant, and sustainable community.” EPA Region 2 is responsible for the execution of EPA programs within the states of New Jersey, New York, the Commonwealth of Puerto Rico, and the U.S. Virgin Islands. EPA ORD develops knowledge, assessments, and scientific tools that form the underpinnings of the majority of EPA protective standards and guidance. The overall goal of this project was to characterize urban pollution using portable sensors, particularly near roadways. While maintaining a common goal, each partner also had their own objectives (Table 2 and Table 3).

#### 2.1.3. Data Collection

Data collection in The Ironbound community involved the deployment of portable sensor assemblies called Citizen Science Air Monitors (CSAM). CSAM units measured nitrogen dioxide (NO_2_) and particulate matter (PM) simultaneously. NO_2_ is a highly reactive gas that can irritate the lungs and contribute to bronchitis, pneumonia, and other respiratory impacts [19,20]. NO_2_ occurs naturally in the atmosphere, as well as through man-made processes such as power plants, industrial processes, and fuel combustion, forming quickly from automobile emissions; elevated concentrations are often found near roadways [21]. PM consists of particles such as soot, smoke, dirt, and dust occurring in various sizes. PM can adversely affect breathing and aggravate respiratory and cardiovascular problems and a host of other issues [22], with the greatest impacts from the smallest particles, including those less than 2.5 microns in diameter (PM_2.5_) [23]. In addition to NO_2_ and PM_2.5_, CSAM units also recorded temperature and relative humidity (RH) with measurements recorded in 5-min intervals.

**Figure 1:**
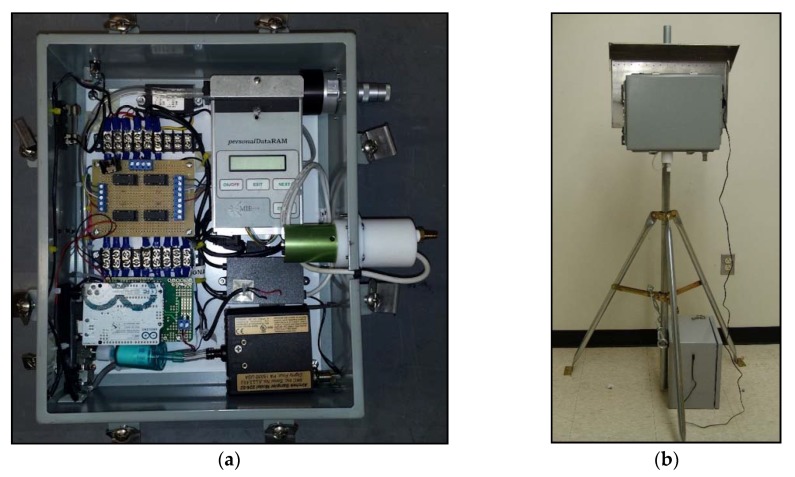
(**a**) Interior of Citizen Science Air Monitors (CSAM) air quality sensor assembly; (**b**) Tripod mounting system with battery attachment for outdoor use.

The self-contained CSAM units included a CairPol CairClip NO_2_ sensor, a Thermo Scientific personal DataRAM PM_2.5_ sensor, and a Honeywell temperature and RH sensor (Figure 1a,b). NO_2_ was recorded in parts per billion (ppb); PM_2.5_ in micrograms per cubic meter (µg/m^3^); temperature in degrees Celsius (°C); and relative humidity in percent at a given temperature (% at °C). Sensors were housed in a weatherproof container with a lockable access door. Data could be retrieved via removable memory cards or accessed directly through a universal serial bus (USB) port on the installed datalogger. Four CSAM units were moved every 2–4 weeks to different sampling locations between February and July of 2015. CSAM units were either mounted on a tripod outdoors and connected to a battery supply, or placed indoors and plugged into a socket while using a snorkel tube to sample outdoor air through a window. They were not designed to sample indoor air. All four units were collocated with FRM monitors for a one-week period near the middle of the study in order to help correct measurement offsets.

### 2.2. Newport News Project

#### 2.2.1. Location Description

Founded in 1896, Newport News, Virginia is located along the James River and has a long history of port activities, shipbuilding, maritime commerce, military activity, and technological development. In addition to port and maritime operations, Newport News includes major rail lines and yards, interstate highways, and coal-handling facilities. There are four major ports in or around Newport News: The Newport News Marine Terminal, Norfolk International Terminals, Portsmouth Marine Terminal, and the Virginia International Gateway. In 2013, Newport News had a reported population of 182,015 people, 41% of whom were African American, 24% of had obtained a bachelor’s degree or higher, and 15% lived below the poverty level. 

#### 2.2.2. Partners, Goals, Objectives

The primary goal of the partnership was to characterize environmental health stressors and identify potential risk reduction actions (Table 4). Partners for the Newport News project included the Southeast CARE Coalition (SCC), a local community organization; EPA Region 3, headquartered in Philadelphia, Pennsylvania, and EPA ORD; The Sierra Club of Virginia, a non-governmental organization (NGO); The University of North Carolina Capstone Course, an undergraduate self-guided research course; a doctoral graduate from Old Dominion University, who had worked previously with the community; and The Greater Southeast Development Corporation, who had worked with SCC previously and continued working with them outside of this particular project. 

#### 2.2.3. Data Collection

Data collection for the Newport News project included a combination of online databases, research-grade (i.e., non-regulatory) air quality models, and local and expert knowledge. Portable sensor measurements were not a part of this study. Prior to the formation of the partnership, SCC hosted town hall environmental health symposiums throughout the Southeast Community, where participants were asked to list and rank their environmental concerns. The partnership utilized this prior information to target research activities, iteratively refining a list of environmental health issues on which to focus data analysis and risk reduction options. 

## 3. Results

### 3.1. Newark

Sensor measurements helped to understand overall conditions for the area, as well as possible high-concentration areas, such as near roads. However, definitive results were difficult to ascertain. Newark measurement collection occurred over several months, from February to July of 2015, with four sensor units being moved every 2–4 weeks between sites, with beginning and end times not always synchronized. Occasional technical malfunctions in certain units limited data collection and contributed to uncertainty about their resulting measurements. Snorkel tubes sampled outdoor air for the units kept indoors, which also contributed to uncertainty. Presented here are a summary of measurements and snorkel considerations, and collocation results with data correction approaches. Results are intended to capture the challenges of translating citizen science results into quantifiable impacts and follow-up actions. 

#### 3.1.1. Data Summary

A summary of recorded measurements is presented in Table 5, including locations and CSAM unit designations, time periods of placement and number of records, snorkel use, data quality flags, and mean values for PM_2.5_ and NO_2_ concentrations, temperature, and relative humidity. Kaufman et al. [18] includes a geographic distribution of measurements. For all time periods and CSAM units (excluding the single day of measurements on 30 July), mean PM_2.5_ ranged between 10.4 and 20.1 μg/m^3^; and 90th percentile values, between 10.9 and 31.8 μg/m^3^. Overall mean PM_2.5_ was 14.5 μg/m^3^; overall 90th percentile mean was 20.3 μg/m^3^. For reference, it must be acknowledged that these CSAM results are not comparable to federal standards for several reasons (technology, quality control, calibration, calculation methods, etc.), ambient standards for PM_2.5_ are 35 μg/m^3^ for a 24-h average, and 12 μg/m^3^ for an annual average. Mean NO_2_ ranged between 4.0 and 47.2 ppb; and 90th percentile values, between 8.1 and 87.4 ppb. Overall mean NO_2_ was 18.9 ppb; overall 90th percentile mean was 40.4 ppb. Like PM_2.5_, federal standards are not directly comparable; however, for reference, NO_2_ ambient standards are 100 ppb for a 1-h average, and 53 ppb for an annual average. As expected, because of its dependence on local sources, NO_2_ demonstrated greater variability between sites compared to PM_2.5_.

**Figure 2:**
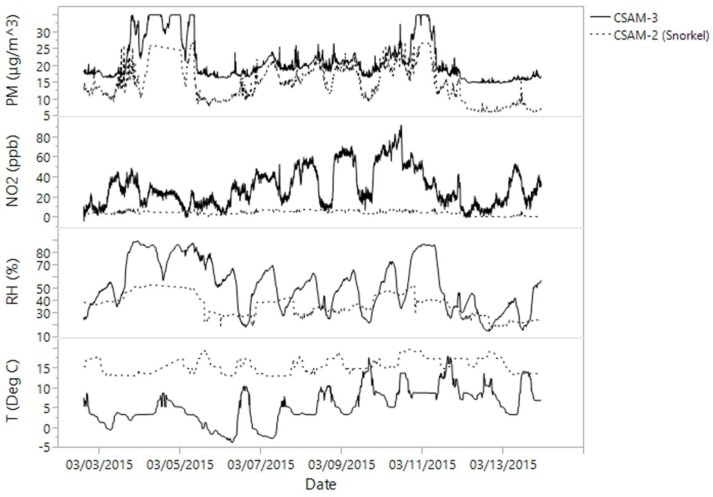
Comparison of two CSAM units with contemporaneous measurements, one with a snorkel tube and one without (not collocated in space).

Data were marked with a green, yellow, or red flag based on good, questionable, or bad data quality, respectively. Green meant that no known issues were found; yellow referred to technical issues or measurement abnormalities; and red typically meant that no measurements were collected because of technical malfunctions. Red flags represented technical failures that precluded measurement collection. Most yellow flags relate to the snorkel assemblies, suggesting leakage of indoor air into the snorkel. Figure 2 shows a comparison between two units at different locations for the same time period, one with a snorkel tube and one without. Snorkel air temperatures recorded slight variations throughout the day compared to the outdoor unit, the snorkel unit hovering closer to room temperature than outdoor temperatures; however, minor variations based on outdoor patterns do appear. Snorkel RH also did not reflect outdoor diurnal patterns and showed minimal variability due to outdoor conditions. Snorkel PM_2.5_ did reflect outdoor variability, but recorded consistently lower values, possibly suggesting attenuation through infiltration and sampling of indoor air. NO_2_ showed no variation and recorded near-zero concentrations for the entire period, suggesting that outdoor air was not sampled. Most snorkeled values reflected these same patterns, and were flagged for potential data quality issues, which resulted in over half the samples having questionable quality.

#### 3.1.2. Collocation and Sensor Corrections

**Figure 3:**
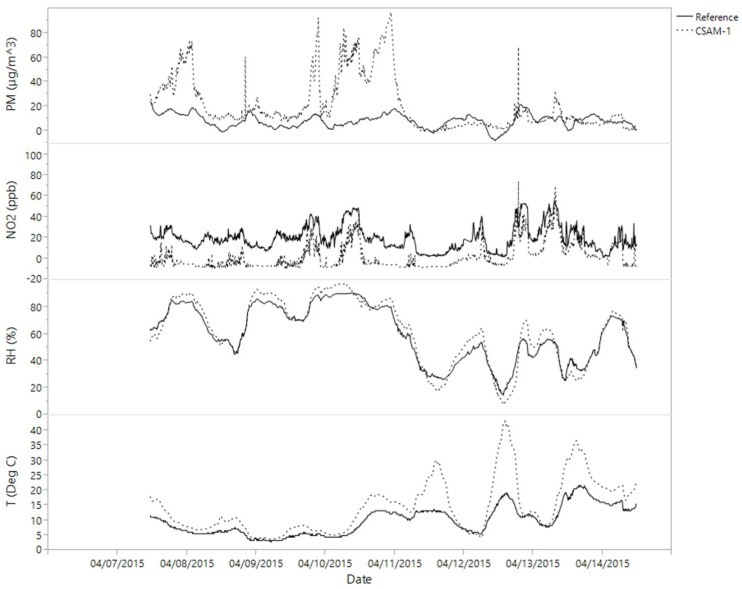
Comparison of one CSAM unit with federal reference monitors during collocation at NCORE station.

**Figure 4:**
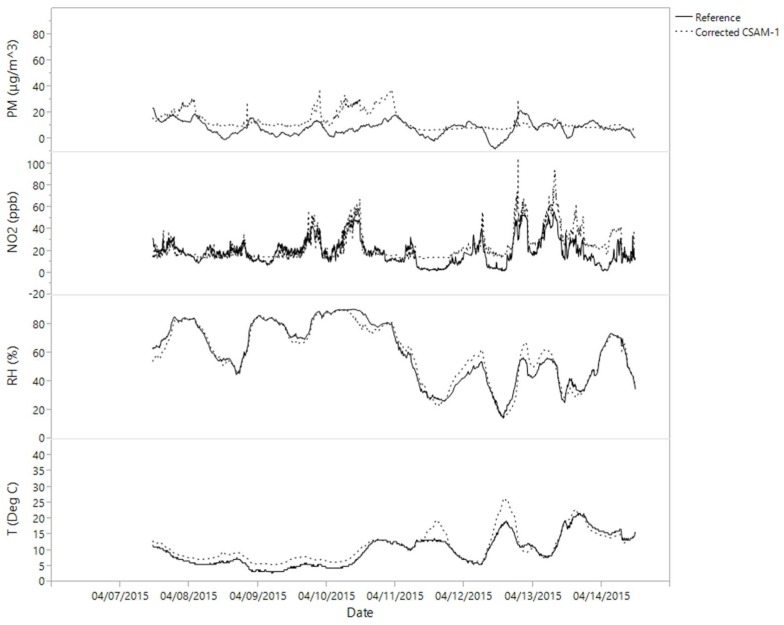
Results of applying regression equation to one CSAM unit based on corrections from federal monitors during collocation at NCORE station.

The four CSAM units were collocated for seven days (7–15 April) at a New Jersey Department of Environmental Protection (NJDEP) National Core (NCore) station with FRM monitors. Two units experienced pump failures that resulted in sparse data collection. Measurements were used to develop a regression equation for each CSAM unit to adjust its measurements. Even though regressions were based on one week of collocation, regressions were applied to the entire dataset. Figure 3 and Figure 4 show an example of a CSAM unit before and after the regression adjustments, respectively. Relative humidity incurred the least adjustment, agreeing well between CSAM and the FRM. Temperatures agreed well except for maximum excursions, which decreased with adjustment. CSAM represented diurnal NO_2_ trends well, but with consistently lower concentrations, which increased to FRM values after adjustment. CSAM PM_2.5_ were generally greater than FRM, especially for maximum values, and diurnal patterns were not as clear or pronounced in the CSAM measurements. These corrections demonstrate the value of collocation for citizen science projects, especially for emerging sensor technologies.

#### 3.1.3. Next Steps

Methods and results were presented at a public forum to characterize measurements and discuss potential next steps. This meeting represented the culmination of the project, so next steps would involve local community partners and how they wanted to proceed. Two primary concepts were conveyed during the report-out: technical abilities of the sensors and context for the measurements. Technical concepts included sensor capabilities and examples of how CSAM and FRM measurements differed. Context included explaining the limitations of the measurements in terms of deducing health impacts and the inability to compare CSAM results directly to regulatory standards. Instead, average measurements were compared to non-regulatory annual averages for 10 different cities, which also was not a direct comparison, nor was it a comparison to regulations. 

Once sensors and context were discussed, participants contributed to possible next steps. The audience provided several suggestions, including a water quality assessment; incorporating a broader network of sensors; evaluating the impact of the nearby international airport; assessing cumulative risks; strengthening collaborative research opportunities with local, academic, government, and NGO partners; targeting sensor measurements based on high-concentration areas; and a health-based (e.g., cancer cluster) epidemiological approach. The depth and breadth of considerations exemplifies the power of local knowledge to contribute to targeted solutions, and the capacity of non-scientists to understand complex information if presented transparently and in an appropriate context.

### 3.2. Newport News

#### 3.2.1. Data Summary

The Newport News partnership collected and analyzed local environmental health information, with an emphasis on potential impacts from port activities, goods movement, and transportation, focusing specifically on port operations, coal piles, shipbuilding, toluene, interstates, asthma, brownfields, food deserts, wastewater treatment facilities, stormwater and sewer line breaks, polychlorinated biphenyl (PCB) contamination, and susceptible and vulnerable populations. Interstate traffic led to concerns about ozone, particulate matter, acetaldehyde, acrolein, benzene, 1,3 butadiene, formaldehyde, diesel particles, carbon monoxide, nitrogen oxide (NO_X_), sulfur oxide (SO_X_), and volatile organic compounds (VOCs). Coal holding facilities led to additional concerns about air quality (see EPA [24] for more information). A list of industrial and traffic-related pollutants and their potential impacts was compiled for stakeholders (Table 6). Exposure scientists clarified that this summary was not conclusive of exposure-health associations. To facilitate risk mitigation efforts, a further table was compiled about potential actions, including information for each environmental health issue, quantification metrics, data sources, and potential risk-reduction actions (Table 7).

#### 3.2.2. Next Steps

The Newport News project culminated in an EPA summary report, including environmental health issues, data summaries, and potential next steps [24]. SCC and EPA Region 3 continued working together after the project to provide training and continue education/outreach and mitigation options. The City of Newport News considered the community work and concerns that came out of SCC’s actions, and identified five key priorities:

1. Increase Trust—Engage local groups to reduce mistrust between them and the city, and assist city employees to improve their comfort level of working with community-based organizations.
2. Encourage Business Development—Secure Housing and Urban Development funds and create an environment for small businesses to open and thrive and make the city more livable.
3. Transportation—Evaluate quick, reliable transportation options to shuttle workers to jobs to facilitate local employment and create more livable circumstances in the community.
4. Promote Home Ownership—Develop a rental rehabilitation and rental inspection program; explore an education program to promote relationships between banks and borrowers; explore a demolition program to remove old infrastructure and rehab vacant lots.
5. Infrastructure—Promote building a new grocery store; improve natural landscapes by planting trees.

## 4. Discussion

One limitation of this study is that no formal questionnaires or interviews were performed to specifically ask participants for lessons and FAQs. Other studies that included online questionnaires [25], semi-structured interviews [26], or a combination of both [27] have an advantage of quantifying results to provide weights and relative importance of responses, as well as to have a comprehensive and consistent response strategy. While the projects presented here and reported in Table 1 did not include these survey mechanisms, they did follow a consistent and structured step-by-step approach (outlined in Methods) similar to the rubric to evaluate citizen science programs presented by Tredick et al. [28]. As part of federal research program standards, rigorous notes, and regular deliverables (summaries, graphs, tables, etc.) were necessary for each project, thus providing thorough, comparable materials from which to draw conclusions. In addition, local community stakeholders provided regular feedback during project formulation stages that contributed to these materials, and EPA Regional and Federal personnel who work closely with local and community organizations and agencies reviewed the findings and provided feedback. 

### 4.1. Decision-Making: People Versus Data

Empowerment has two connotations: the first is to grant decision-making authority, another is to build strength and confidence to take control of a situation. Between these two endpoints is a gray area into which citizen science EHAs could fall, providing participants with an understanding of detrimental situations without the legal authority to enforce mitigation. Unlike regulatory standards, citizen science measurements and data analyses are subjective forms of legal tender—only those who accept them will place value in the results [29]. The more substantive the research plan, quality assurance requirements, citizen training, expert input, and peer review a citizen science plan has, the more likely the data will be accepted as objective reality by outsiders [30,31]. Groups may not be able to afford projects like the ones outlined above; for example, CSAM units cost approximately $6000–$7000 for parts and professional assembly. However, outside of the sensors, time was largely voluntary or part of EPA researchers’ official duties, and data mining drew from free, publicly available sources. Limitations in terms of technical abilities to access and analyze data may prove prohibitive, yet limited financial resources can go a long way given the appropriate expertise and a thoughtful research design that is targeted to the decision-making audience.

Multi-partner collaborations could contribute to a *perception of empowerment* as well as a *perception of adversity*, based on whether stakeholders consider themselves insiders or outsiders to accomplishing the goal. If a community is partnering with a government agency, as occurred in the above examples, there could be a *de facto* expectation that policy changes and mitigation actions will ensue; that partnering with a federal agency empowers the community with legal authority to take action. In some projects listed in Table 1, the communities expressed excitement and willingness to partner with the EPA, yet this enthusiasm waned when community expectations of health improvement did not directly follow the relatively limited data collection effort. This perception of empowerment was not realized. Similarly, local health and environmental agencies, and private/commercial interests, could retain a perception of adversity, being cautious to pursue a collaboration should there be expectations that local agencies implement new policies or relinquish control, or commercial interests undergo enforcement and compliance actions or change business practices, based on the results, especially if the federal government’s main partner is the local community. Conversely, these perceptions could reverse depending with whom the federal government partners. Several projects in Table 1 had to address this dynamic, and in most cases, potential opponents to the goal were not engaged, possibly to the detriment of long-term success. Perceptions of both empowerment and adversity can be a barrier to building partnerships with precisely the parties needed to implement change. 

Through this lens of data and decisions, three lessons and multiple FAQs came into focus. While locally-based, citizen science EHAs may share certain commonalities, each is unique in its people, problems, and approaches [32,33]. Sometimes the greatest environmental risk is not always the highest priority because of local values or resources. Thus, lessons and FAQs presented here are based on commonalities from multiple projects, but tailorable to each unique situation. 

### 4.2. Three Lessons

#### 4.2.1. Partners May Share Goals, but Not Objectives

In a multi-partner EHA, it is quite possible that each partner will agree on the same goal, but have a different set of objectives, such as for the Newark project (Table 2). For the purpose of this research, a goal is defined as a long-term aspiration or aim, and could be nebulous and difficult to measure; examples include, “Improve community health,” or, “Protect vulnerable populations from environmental stressors.” Alternatively, objectives are concrete, measureable achievements that can be attained after certain steps, typically with short- or medium-term timeframes; examples include, “Host town hall meetings to educate community on emission sources,” or, “Measure air quality to characterize near-road hotspots.” All partners should fully understand the exact goals and objectives of data collection, answering, “Why, *exactly*, are we doing this?” Or, as a community leader aptly described, to “work with intention.”

Objectives should be as specific and unambiguous as possible. The Newark and Newport News approaches were quite different—one being sensor-driven and the other focused on existing data—and yet the community stakeholders had similar objectives: to improve community health through various actions, including education/outreach, policy change, and emissions mitigation. However, the Newark sensor project experienced technical challenges and translation of results to action. Also, in Newark, EPA experts provided the community with sensors and sensor expertise, but the community wanted measurements to assess health impacts and support mitigation actions. There was a disconnect between results and solutions that never fully resolved. In the end, the community was given a thorough summary of all the technical aspects, and retained ownership of the measurements, but follow-through for actions was primarily their responsibility. In Newport News, the initial approach was quite different. First, the project never planned to use sensors because of the project funding and research logistics. Also, the primary goal was to test and apply an assessment process and new GIS and data mining methods, so education/outreach was a primary goal. There were also several complementary efforts going on in the EPA Region to continue working with the Newport News community, so follow-up actions were also supported by the professional network rather than project results. For successful EHAs, goals and objectives should be delineated for each group in the partnership, and roles and responsibilities for each group and individual [34]. These should be discussed early and revisited at planned intervals throughout the project. Stakeholder engagement involves consideration of each group or individual and determination of whether their objectives are supported by the data collection and assessment efforts. 

#### 4.2.2. Graphs Do Not Make Decisions, People Do

Data and measurements alone do not enact change; people do [35,36]. There is no established method to translate citizen science sensor measurements into regulatory action, for example [18,30]. Arguably, the best use of data in these cases is to develop a hypothesis-driven narrative that uses data as evidence of potential emissions, exposures, and impacts. The exact same data is capable of either substantiating bias or changing minds, depending on how they are presented and who is looking at them. For example, average PM_2.5_ concentrations in Newark did not exceed national standards for PM, but did place the community within the top 10 cities with high annual average PM_2.5_. However, measurements and calculations for the national standards and the citywide approach were not equivalent to the project design, so were not representative comparisons, but at least provided some context. Newark data were suggestive but not conclusive. Would community members take results as proof of their concerns? Would regulators cast doubt on the measurement quality? Would both groups agree to a more detailed study or precautionary actions, or conversely, conclude it was all a waste of time and effort? 

The answer to combatting subjectivity is simple: engage all decision-making authorities early in the process; plan a scientifically-defensible, peer-reviewed research plan; and develop early and ongoing consensus on how the group will respond to results (e.g., “If we find this, then we’ll do that”). The answer is simple; implementation is not. In Newark, local government authorities were not engaged early in the process, yet it became clear that partners were interested in presenting results to influence local policies. The partners for Newport News agreed on a general characterization approach and not political action, so decision-making authorities were not involved. Neither project considered the specific question of how data were to be used after collection to influence decisions or next steps.

Two things that any project can do to help ensure relevance are: (1) develop a scientifically sound research design, and (2) know your audience. Sound strategies include sensor collocation and calibration; expert training; representative spatial and temporal scales; and regular quality control checks [30,36]. The EPA provides sampling strategies on their Citizen Science Toolkit webpage (https://www.epa.gov/air-sensor-toolbox). Knowing your audience means researching who would be involved in decision-making based on the data and knowing how to communicate to them. For community members, how will data be presented in a meaningful way? For local agencies or policy makers, what are their data quality requirements? A sound strategy and a practical approach will help ensure that time, effort, and passion are not wasted. 

#### 4.2.3. Measure Success and Manage Expectations Regularly and Often

Groups are composed of individuals who have opinions on how things should run, who is in charge, and what things mean. In a collaborative EHA, question and clarify expectations constantly to determine whether the data will support project goals and objectives. For example, researchers have a proclivity to assume that *more research is necessary* is a reasonable conclusion to a project. After more than two years of developing and implementing a citizen science project in Newark, this conclusion would have been unacceptable to all partners as the sole result. EHAs evolve through time, so adaptive management is necessary to stay focused on the original goal or develop a new one when applicable. Set a few reasonable, attainable objectives, and when they are met, celebrate them. Advertise success; market it as justification for the work; use it to incentivize participation. Success is the fuel that keeps collaborations running smoothly.

These projects offered benefits for all participants, yet neither project led directly to improvements in health, reductions in contaminant emissions, or changes in policy. However, both projects led to long term collaborations with city and state agencies to promote environmental health, and while unexpected, were welcome outcomes. Since Newport News was an MVD community, several other projects were similarly occurring in the area, and ultimately the community and city worked together to develop a common understanding and potential future directions. In Newark, the sensor results were not used to assess health impacts or identify local hotspots. However, the empowerment of a community conducting such an intensive sampling campaign led to discussions with the city of Newark to engage and promote environmental health activities and bring greater awareness to local community concerns. While results from neither project directly supported change, the process and empowerment eventually promoted environmental health efforts through enhanced collaboration and awareness. 

### 4.3. FAQs

Over 30 FAQs for citizen science EHAs were developed based on a compare and contrast of each step of the assessment process for the Newark and Newport News projects, as well as for several from Table 1. Lead organizers for the citizen science projects in Newark and Newport News, as well as for Kansas City, and HIA and EJ experts on locally-based projects provided input and feedback to the FAQs. Each step included interactions with various partners, including communities, local/state agencies, and federal representatives. A summary of FAQs provided in Table 8, and detailed considerations for each question are included in Supplemental Materials (Table S1). Input from commercial/industrial interests is admittedly under-represented. These FAQs are primarily intended for community residents and local stakeholders. Additional FAQs could be developed to address commercial interests and/or local political stakeholders. Since each project is unique, FAQs should be tailored to specific needs, such as by selecting a subset, using language, and crafting responses appropriate for local stakeholders. 

## 5. Conclusions

When the environment includes everything that a person senses—sees, feels, hears, touches, breathes, drinks, eats, smells—and impacts range from toxic health responses to emotionally-induced physiological stress, then EHAs can quickly escalate in terms of potential stressors, salutary factors, and impacts, as well as in the number and variety of stakeholders and partners. While not all EHAs have such broad considerations, retaining focus can prove challenging in the context of so many possibilities. These lessons and FAQs are intended to support citizen science EHAs given the complexities of both environmental health and interpersonal relations.

Characterization alone is rarely the objective of citizen science EHAs. Based on the partnerships described above, for example, community members are interested in actions, solutions, and change; regional authorities, in supporting state-wide initiatives; local agencies, in balancing engagement with pragmatism; federal researchers, in developing transferable methods for national use; and commercial/industrial interests, in acknowledging their role in the community while maintaining a practical self-interest. Acknowledging a mutual goal as well as each group’s specific objectives embraces this diversity and facilitates project success and longevity. 

Underlying any assessment should be the question, “Why?” Why are we doing this? What do we expect to happen as a result of this data collection, interpretation, and communication? EHAs should be tailored to the audience that ultimately helps to improve environmental conditions, strengthen salutary factors, and build strong partnerships. Measurements, graphs, and model simulations alone do not enact change, people do. When developing a research plan, for example, consider reaching out to a local academic institution for review and engagement, or to a public health agency to ask about quality assurance requirements, or to communication and visual arts students to design brochures. In an EHA, be open to asking: who are the clients (e.g., local policy makers); what are you trying to sell them (e.g., mitigation incentives); what do you get in return (e.g., improved environment; decreased impacts). Goal-oriented EHAs strengthen both aspects of empowerment, allowing partnerships to both understand local problems as well as to do something about them.

## Figures and Tables

**Table 1:** Description of locally-focused projects related to environmental health assessments facilitated by the Environmental Protection Agency (EPA) Office of Research and Development (ORD) (does not represent totality of ORD projects; Chicago includes two separate projects). Projects with (*) are covered in this paper.

General Area	Summary
Detroit, MI; Holyoke, MA	Developed GIS community mapping process and maps
Milwaukee, WI; Chicago, IL; Port Arthur, TX	Environmental Justice indicators were compiled and displayed for comparison at four spatial scales: local, city, county, and state
Charleston, SC	Air quality characterization of freight movement operations related to port expansion activities
Birmingham, AL	Characterize historical, current, and future environmental conditions, including blight, proximity to industry and rail yards, lack of health care and retail, unemployment, and decline in residential population
Chicago, IL	Analyze and interpret multiple stressors in communities adjacent to landfills, industrial areas, brownfields, and interstates
Newark, NJ *	Conduct a citizen science air quality sensor project to identify trends and high concentration areas
Newport News, VA *	Assess potential impacts of environmental stressors with additional social, economic, demographic factors that relate to community well-being
Kansas City, KS	Measure local air quality in neighborhoods surrounded by multiple emission sources, including industry, diesel trucks, rail facilities and major highways
Wichita, KS	Compile metrics and actions to address multiple issues, including infant mortality, asthma, industrial emissions, susceptible and vulnerable populations, awareness, and life expectancy
Portland, OR	Assess children’s potential exposure to particulate matter and emissions during school bus commute and school attendance near busy roads

**Table 2:** List of partner groups and respective objectives for the Newark, Ironbound community project.

Partner	Objectives
ICC	- Characterize near-road/near-source high-concentration areas - Consider public housing adjacent to both ground-level truck routes and elevated rail lines and highways
EPA Region 2	- Develop a standard operating procedure (SOP) for portable sensors - Consider options for sensor loan program for public use
EPA ORD	- Develop methods for EPA citizen science air sensor toolbox (https://www.epa.gov/air-sensor-toolbox) - Assess sources of sensor uncertainty/variability

**Table 3:** List of individual roles and responsibilities for the Newark, Ironbound community project.

Role	Responsibility
ICC Project Manager	- Oversee activities of Community Liaison and Volunteers
ICC Community Liaison	- Conduct outreach to facilitate community involvement in study-related needs such as design, data collection, analysis, and reporting results - Attend training at EPA offices for equipment handling and maintenance
Community Volunteers	- Support study needs including equipment handling and maintenance, data collection and management, community updates - Update partners on progress and challenges
Region 2 Project Coordinator	- Coordinate study activities - Ensure communication between project staff and partners
Region 2 Citizen Science Liaison	- Advise study partners on citizen science issues such as Quality Assurance Project Plan (QAPP) development, etc.
Region 2 Community Technical Support	- Train volunteers on sensor use and data download procedures - Participate in the collaborative site selection process - Trouble-shoot download/maintenance issues as they occur
ORD Principal Investigator	- Manage instrument assemblies and citizen science toolbox - Maintain milestones and documentation - Facilitate interaction between technical requirements and community-specific needs
ORD Technical Advisor(s)	- Provide technical expertise on instrument assemblies as related to project objectives, and input on ORD citizen science toolbox

**Table 4:** List of partners, roles, and responsibilities/objectives for Newport News project.

Partner	Role	Responsibility/Objective
Southeast CARE Coalition	Community Organizer: assess and improve environmental health outcomes in Newport News, Virginia	Generate action to improve community health; assemble, analyze, and disseminate community-specific information on pollutants, risks, and impacts
Greater Southeast Development Corporation	Reduce exposure to toxic pollutants and improving the environment	Engage residents, businesses, academics, non-profit/grassroots, city/state/federal agencies
EPA Region 3	Research collaborator and liaison between community and partners	Provide local support based on multi-state expertise
EPA Office of Research and Development	Research support and method development	Assist with data collection, analysis, interpretation, and communication
Sierra Club of Virginia	Help Coalition stay on target and maintain positive efforts	Plan community events, organize tours, support coalition efforts
University of North Carolina Capstone Class	Student-led undergraduate research to support project needs	Support data collection, analysis, and interpretation
Old Dominion University	Research support and community outreach/education	Research major sources of toxics in the community and the health risk associated with exposure

**Table 5:** Data summary for The Ironbound community citizen science measurements.

Location & Unit.	Time Period	Snorkel Y/N	Flag G/Y/R (Reason)	PM_2.5_ (μg/m^3^)		NO_2_ (ppb)		T (°C)	RH (%)
				Mean	90th Percentile	Mean	90th Percentile	Mean	Mean
L1 U1	12–27 February	N	G	13.1	20.8	41.4	80.8	1.2	53.0
L2 U2	12–27 February	Y	Y (High T; Low ΔT)	13.2	18.8	10.9	26.8	20.6	27.6
L3 U3	12–24 February	N	G	19.4	25.0	18.3	40.2	-1.3	48.1
L4 U4	12–27 February	Y	Y (High T; Low ΔT; PM N/A)	--	--	18.6	38.1	16.1	37.8
L5 U1	3–16 March	Y	Y (High T; Low ΔT)	12.1	17.7	13.8	15.0	17.6	25.0
L6 U2	28 February–16 March	Y	Y (High T; Low ΔT)	14.7	21.5	5.3	8.1	15.6	37.0
L7 U3	3–16 March	N	G	20.9	31.8	27.2	56.2	6.1	55.8
L8 U4	2–16 March	Y	R (Pump Failed)	--	--	--	--	--	--
L9 U1	18–25 March	N	G	10.4	14.8	27.5	56.2	7.8	39.6
L10 U2	17 March–6 April	Y	R (No data)	--	--	--	--	--	--
L11 U3	18–25 March	Y	G	16.6	17.8	8.7	28.4	9.4	25.4
L12 U4	18 March–6 April	Y	R (No data)	--	--	--	--	--	--
L13 U1–4	7–15 April	N	Collocation	--	--	--	--	--	--
L14 U1	21 April–12 May	Y	G	10.8	17.7	38.2	87.4	19.5	34.7
L15 U2	21 April–11 May	Y	Y (High T; Low ΔT; PM N/A)	--	--	15.1	43.4	19.8	42.3
L16 U3	24 April–11 May	Y	G	16.6	21.5	22.0	49.7	13.2	46.9
L17 U1	25 June–10 July	Y	Y (6/29–7/1; 7/5–7/10)	10.9	16.1	27.0	46.2	19.9	53.9
L18 U2	25 June–14 July	N	R (Card taken; battery unplugged)	--	--	--	--	--	--
L19 U3	25 June–14 July	Y	G	16.2	19.5	4.0	16.2	18.3	51.2
L20 U4	16 June–14 July	Y	Y (6/16–6/26; PM N/A)	--	--	5.2	13.4	22.4	63.8
L21 U3	30 July	N	G (single day)	16.6	17.22	1.0	6.9	19.5	62.4

**Table 6:** List of chemicals and potential exposure-response symptoms associated with emissions sources in the Newport News project study area.

Chemical	Potential Exposure-Response Symptoms
*Industry-related*	
*N*-butyl alcohol	Impaired hearing and reduced vision
Xylene (o-xylene)	Eye damage; nausea; poor coordination
Sulfuric acid	Dental effects; pulmonary edema
1,2,4-Trimethylbenzene	Anemia; nausea; skin, eye, throat, and respiratory irritation
Ethylbenzene	Headaches; irritation of mucous membranes
Copper	Anemia; damage to lungs, liver, kidneys, and nasal septum
Manganese	Cough and fever; insomnia; kidney damage; weakness; back pain
Nickel	Sensitizing dermatitis; allergic asthma; potential carcinogen
Chromium	Lung fibrosis; eye and skin irritant; potential carcinogen
Trichloroethylene	Visual distortions; headaches; potential carcinogen in liver and kidney cells; cardiac arrhythmia
Zinc (zinc chloride)	Metal fever, nausea, and cough, which reduces pulmonary function; vomiting; back pain
Toluene	Effects on central nervous system; fatigue; sleepiness; headaches; nausea; irritation of respiratory tract, eyes, throat; dizziness; headache; pregnancy-related developmental effects, including attention deficits
*Traffic-related*	
Particulate Matter 2.5 (PM_2.5_)	Heart attacks; irregular heartbeats; increased asthma symptoms; airway irritation; decreased lung function
Acetaldehyde	Eye, respiratory, and skin irritation; increased blood pressure; decreased respiratory rate
Acrolein	Eye, respiratory, nasal, skin irritation; respiratory congestion
Benzene	Drowsiness; dizziness; headaches; respiratory, skin, and eye irritation; blood disorders; leukemia; reproductive effects; known carcinogen
1,3 Butadiene	Eye, nasal, respiratory irritation; cardiovascular effects; leukemia; known carcinogen
Formaldehyde	Respiratory irritation; coughing; sneezing; wheezing; chest pain; bronchitis; reproductive damage; known carcinogen, including lung and nasopharyngeal cancer
Carbon Monoxide	Reduced oxygen delivery; exacerbates cardiovascular disease and chest pain
Nitrogen Oxides (NO_X_)	Cardiovascular disease; asthma exacerbation; bronchitis; emphysema
Sulfur Oxides (SO_X_)	Bronchoconstriction; asthma exacerbation; cardiovascular disease
Volatile Organic Compounds (VOCs)	Eye, respiratory irritation; liver, kidney, and central nervous system damage is possible depending on specific chemical

**Table 7:** List of environmental health issues and their related quantitative metrics, data sources, and potential risk reduction approaches for Newport News project.

Issue	Metrics	Data Sources	Potential Risk-Reduction Approaches
Ports	- Increased traffic - Air emissions from port activity - Daily imports/exports (Goods movement) - Before/after expansion plan - Water quality, effluent, stormwater, coastal, drinking	- Air quality (AQ) dispersion models - National Emissions Inventory (NEI) - National Air Toxics Assessment (NATA) - Ambient Air Quality Systems (AQS) data - Prior studies - Toxic Release Inventory (TRI)	- Connect with Port Authority - Run AQ models to target impacted areas - Reduce outdoor exposures during high-concentration events - Mitigate indoor sources to reduce cumulative exposure
Coal Piles	- Coal dust pollution - Numbers of piles - Locations of piles - At-risk downwind locations (e.g., schools)	- Prior studies - Coal pier/pile surface area, locations, prevailing wind directions	
Shipbuilding	- Air emissions - Water quality near facility	- NATA, NEI, TRI	
Toluene	- Ambient air concentrations - Sources and emission volumes - Hazardous waste sources	- NATA, NEI - Resource Conservation and Recovery Act (RCRA) sites - Prior studies - AQ models	- Mitigate indoor sources to reduce cumulative exposure - Education on multi-media (e.g., soil) exposures - Biomarker testing to estimate exposure
Interstate	- Traffic patterns and congestion - Asthma and respiratory illnesses	- Local truck counts - NATA, AQS - Near-road AQ models	- Target outreach to - Truck counts - Emission reduction initiatives
Asthma	- Asthma incidences - Missed days of school/work - Hospitalizations - Ambient air pollution	- AQS, NATA - Near-road AQ models - Hospital/school records	- Education and outreach on triggers and exposure reduction
Brownfields	- Decreased property values - Increased crime rates - Aesthetics	- Zoning records - County reports - Green space data - Proximity	- Explore potential for mitigation and greenspace
Food Deserts	- Number of grocery stores - Food stamps/school lunches - Access to produce - Malnutrition; BMI	- USDA food desert maps - Public transportation to stores	--
Wastewater Treatment Facility	- Water quality - Air emissions - Respiratory issues	- NEI, NATA, AQS, TRI - Water quality records	--
Stormwater and Sewer Line Breaks	- Infrastructure - Water quality	--	--
Watershed PCB Contamination	- Beach closings - Concerns about subsistence fishing	- Prior studies - TRI	--
Vulnerable Populations	- Income (poverty) - Education - Race/Ethnicity - Proximity - #/Area of pollution sources - # Children - # Elderly	- EJ Screen	- Multi-lingual educational materials - Engage with community leaders

**Table 8:** Summarized list of Frequently Asked Questions (FAQs) for citizen science collaborative projects. Detailed considerations for each question can be found in Supplemental Materials.

**What Is the Purpose of the Study?**
Describe the problem the study is addressing, and the ultimate benefit the study will have for the community as well as its science, research, and/or programmatic purpose.
Who is leading the study?
Which community is involved in the study?
Why this community? (How was the community selected?)
What previous studies have been performed in this community/area? How does this study complement those or how is it different? What is the added value of this work?
What stakeholders are involved in the study?
What level of participation is the community being included in?
How is the community involved in the study process?
How long will the study last? When will the study start and end?
What is the benefit to the community overall and to individuals in the community? How does this benefit the community? What will the community get out of this study?
**What is the end goal of the study? How will the results be used?**
What are the results intended to show? Are sensors supposed to capture absolute concentrations, relative differences (i.e., hotspots), spatio-temporal trends?
How does this study benefit the community? What will the community get out of this study?
How can you (as a community member) use these tools, data, and sensors to gather information on environmental health risks to you and your community? (What is in it for you?)
**How will information be shared with the community?**
Where and how will information be shared with the community?
Ask community: What is the best method for sharing information with you?
How will private or sensitive information be protected?
How can the community keep up with what is going on with the project? Where can I find updates?
How will concerns of community members about the data or results be addressed?
How can community members contact the study team if they have questions about the study?
**What topics and research methods does the study include?**
**Pollutants being considered**
Which pollutants are included in the study? Which are being measured or modeled?
Are there regulations for these pollutants? If so, then why are we measuring it?
Where do these pollutants come from? What are the major sources of these pollutants?
Where are these pollutants found—in the air, water, soil, food, household?
Why are other pollutants not being investigated?
**Types of Measurements, Models, and Results**
What sensors are being used to measure these pollutants? What are the benefits and limitations of these sensors
Which computer models are being used to estimate pollutant concentrations? What are the benefits and limitations of these models?
Will this study pinpoint who is producing these pollutants?
Are these measurements and models the same ones the EPA uses to enforce regulations, such as the Clean Air Act? Can results from measurements or models be used to make the sources reduce their emissions or output of these pollutants?
Will the community or public have access to the results of this study? Who ‘owns’ the data that is collected? Where will the results be kept?
How will results be reported—as a written report, charts and graphs, maps?
**Public Health**
Which health effects are associated with these pollutants?
What are considered ‘high’ or ‘dangerous’ concentrations of these pollutants? What happens if we measure high concentrations? Does this mean my health is at risk, or that we can take legal actions to reduce the amount of pollution?
What are different ways to reduce exposure to pollution?
Which other things might be present in the community that could influence my health? Are these being considered?
**Other Studies or Analyses?**
What other studies or analyses have been conducted in the vicinity of our community?
What are the results of these studies/analyses?
How scientifically grounded are these studies/analyses?

## Supplementary Materials

The following are available online at http://www.mdpi.com/1660-4601/15/5/960/s1, Advice and Frequently Asked Questions (FAQs) for Citizen-Science Environmental Health Assessments.
