# Supplementary material for: Advice and Frequently Asked Questions (FAQs) for Citizen-Science Environmental Health Assessments
Source: Int J Environ Res Public Health. 2018 May 11;15(5):960. doi: 10.3390/ijerph15050960 (PMC5981999; doi:10.3390/ijerph15050960)
Supplement: Supplementary file 1 [file ijerph-15-00960-s001.pdf]

Advice and Frequently Asked Questions (FAQs) for Citizen-Science Environmental Health Assessments

Supplemental materials for Table 8

Table S1. Detailed considerations for each Frequently Asked Question (FAQ) for citizen science collaborative projects.

Question
**What is the purpose of the study?**
**Describe the problem the study is addressing, and the ultimate benefit the study will have for the community as well as its science, research and/or programmatic purpose.**<br>• Why here? Why now? – why are you measuring these specific things right now?<br>• Be specific – be careful of broad goals of "improving health" or "building sustainability" or working with everybody to solve everything<br>• Good place to state what project will NOT do – not for enforcement/compliance; not source apportionment; not health study; will not force legal/regulatory action<br>• Measure what? Report to whom? What are the specific, intended or expected outcomes?<br>• Build capacity, educate, inform, enlist local agencies/policy makers – worthy goals as well<br>• Does this study support a policy, enforcement, or compliance?
**Who is leading the study**?<br>• Name the lead agency(ies) or committee/coalition including names of member organizations
**Which community is involved in the study?**<br>• Name the community, being as specific about neighborhood or demographics as possible
**Why this community? (How was the community selected?)**<br>• Describe the process of selection including who was involved in the selection
**What previous studies have been performed in this community/area? How does this study complement those or how is it different? What is the added value of this work?**
**What stakeholders are involved in the study?**<br>• Name organizations or individuals involved in any aspect of the study
**What level of participation is the community being included in?** For example, see IAP2 Spectrum of Public Participation
**How is the community involved in the study process?**<br>• Describe how the community is involved in each phase of the study<br>• Study questions and goals<br>• Study design<br>• Data collection<br>• Data analysis<br>• Review of results<br>• Dissemination of results
**How long will the study last? When will the study start and end?**<br>• Describe in terms of when community involvement will begin and when results will be available
**What's the benefit to the community overall and to individuals in the community? How does this benefit the community? What will the community get out of this study?**

What is the end goal of the study? How will the results be used?

What are the results intended to show? Are sensors supposed to capture absolute concentrations, relative differences (i.e., hotspots), spatio-temporal trends?

- Is the goal to characterize the situation and develop potential risk/exposure reduction actions, and identify who would be involved with those and how to build consensus? Or is it simply to characterize pollution sources and that's all?

How does this study benefit the community? What will the community get out of this study?

- Describe the specific benefits the community will receive
- Mention relation to solutions and/or health impacts resonates with public communities
- Keep in mind: 1) some communities have been studied a lot and data supported no real changes (i.e., paralysis by analysis), and 2) they're skeptical of the returns of research projects, which is why researchers have become alienated from working with them
- Put yourself in the shoes of the community, as well as the local agencies and academics – residents want results and change; agencies are cautious but looking for support and alternative solutions outside of more regulations; and academics often support both research and activism

How can you (as a community member) use these tools, data, and sensors to gather information on environmental health risks to you and your community? (What's in it for you?)

How will information be shared with the community?

Where and how will information be shared with the community?

- Describe whether information will be shared through reports, presentations, websites, emails, etc.

Ask community: What is the best method for sharing information with you?

- How will results be reported – as a written report, charts and graphs, maps? Refrigerator magnets with contact info, for example

How will private or sensitive information be protected?

- State whether any information is being collected about individual community members or their neighborhoods, and how this information will be handled, such as anonymity or confidentiality, IRB review, review by a community advisory or oversight committee

How can the community keep up with what's going on with the project? Where can I find updates?

- Managing expectations throughout the project is important
- How will stakeholders keep up to date on the progress? Through what mechanism?
- Provide regular updates about progress, with constant references to the deliverables and expected outcomes – make sure everyone is on the same page with what will happen at the conclusion of the work

How will concerns of community members about the data or results be addressed?

- Anticipate concerns that community members might have and prepare how to address them

How can community members contact the study team if they have questions about the study?

- Give contact information for someone who will handle questions about the study

What topics and research methods does the study include?

Pollutants being considered

Which pollutants are included in the study? Which are being measured or modeled?

- Good place to include hyperlinks to EPA information on these pollutants

Are there regulations for these pollutants? If so, then why are we measuring it?

- Good place to explicitly state how, for example, the PM standards are calculated (3 year, 95th percentile, constant calibration/maintenance, etc.) and how citizen science results differ from that method

Where do these pollutants come from? What are the major sources of these pollutants?

- Can state in general the potential sources, but remember that folks will fill in the blanks for which local sources might be producing them – if you say “paper mills” then they’ll think that you’re measuring emissions from the local paper mill
- State whether or not you’re doing source apportionment – are you identifying the local contributing sources, or measuring ambient air quality, which is a mixture of all local sources?

Where are these pollutants found – in the air, water, soil, food, household?

- Make people aware of the complexity of pollution levels
- Here or somewhere else, let people know that concentrations can vary in space and time, and that measured outdoor concentrations, for example, are not the same as exposure levels

Why aren’t other pollutants being investigated?

- Can mention that certain pollutants are representative of others as well
- Another place to discuss/reinforce the limitations of the study

Types of Measurements, Models, and Results

What sensors are being used to measure these pollutants? What are the benefits and limitations of these sensors?

- Benefits include relative differences, spatial/temporal trends, and targeted areas
- Limitations include non-federal/regulatory approaches, non-stationary for long term averages, potential calibration issues
- Other types of limitations include non-source apportionment, not representative of personal exposures, cannot be used for enforcement/compliance

Which computer models are being used to estimate pollutant concentrations? What are the benefits and limitations of these models?

- Same as for sensors but for models
- Other benefits include high resolution for exploratory analysis
- Other limitations include being representative only under certain conditions

Will this study pinpoint who is producing these pollutants?

- Folks can tend to think that results could be used to attribute blame to emission sources, and then put pressure on policy makers to do something about it
- This answer should be developed in a way that even the emission source stakeholders would be interested in the results

Are these measurements and models the same ones the EPA uses to enforce regulations, such as the Clean Air Act? Can results from measurements or models be used to make the sources reduce their emissions or output of these pollutants?

- Good place to explicitly state how, for example, the PM standards are calculated (3 year, 95th percentile, constant calibration/maintenance, etc.) and how these results differ from that method

Will the community or public have access to the results of this study? Who ‘owns’ the data that is collected? Where will the results be kept?

- If, for example, the community or local agency wanted to publish or make a news announcement using the data, can they?

How will results be reported – as a written report, charts and graphs, maps?

- Again, what can people expect from the results?

- This is also good for researchers to start developing an analysis plan when the data starts to roll in – have a clear, early idea of what you expect to see and what you plan to do with it
- If people are supporting this study, then they probably expect to see “bad” results – bad air, bad exposures, links to health effects – how will the data be displayed to answer those questions? To what will results be compared to determine whether conditions are bad or not? What if conditions are not that bad?

Public Health

Which health effects are associated with these pollutants?

- Link to CDC-ATSDR, for example, for available information – use what is already out there and referenceable
- Which health effects and how were they determined?
- Are they based on chronic or acute exposures? Human or animal studies? Epidemiological or tox pathways?
- What does this study have to do (or not) with determining health effects?

What are considered ‘high’ or ‘dangerous’ concentrations of these pollutants? What happens if we measure high concentrations? Does this mean my health is at risk, or that we can take legal actions to reduce the amount of pollution?

- This question can be rephrased, but not ignored
- Most folks in a study like this will expect to see “bad” results – they expect to see high concentrations or negatively-impacted areas. What happens if results do or don’t reflect that?
- What are the next steps if high concentrations are found? What avenues are available from policy, research, or local actions?

What are different ways to reduce exposure to pollution?

- Emissions vs. exposure reduction
- Stakeholders – public residents, emission sources, local agencies, academics – should be made aware of the different avenues to help reduce exposures and hazards, especially from a non-regulatory standpoint
- Emissions reduction, exposure reduction (e.g., air filters), education/outreach, and building resilience (health care, nutrition, greenspace) are potentials as well

Which other things might be present in the community that could influence my health? Are these being considered?

- The community has local knowledge about what’s going on around them. They should know that other factors can be influencing their health as well – personal behaviors, stress, chemical mixtures, nutrition, pre-existing conditions, etc. should be noted
- Also, positive aspects can be mentioned as well – social/religious networks, outreach programs, and community pride can be leveraged to help to improve health

Other Studies or Analyses?

What other studies or analyses have been conducted in the vicinity of our community?

What are the results of these studies/analyses?

How scientifically grounded are these studies/analyses?
